# Efficacy of BRAF/MEK-inhibitor therapy for epithelioid glioblastoma with a novel BRAFV600 mutation

**DOI:** 10.1186/s40478-024-01834-8

**Published:** 2024-08-06

**Authors:** J. Steininger, C. Buszello, R. Oertel, M. Meinhardt, S. Schmid, K. Engellandt, S. Herold, S. Stasik, A. Ebrahimi, B. Renner, C. Thiede, I.Y. Eyüpoglu, G. Schackert, S. Beissert, F. Meier, J. Radke, D. Westphal, T. A. Juratli

**Affiliations:** 1 Department of Dermatology, University Hospital Carl Gustav Carus, Technische Universität Dresden, Fetscherstraβe 74, 01307 Dresden, Germany; 2 Department of Neurosurgery, University Hospital Carl Gustav Carus, Technische Universität Dresden, Dresden, Germany; 3 Institute of Clinical Pharmacology, Medical Faculty, Technische Universität Dresden, Dresden, Germany; 4 Institute of Pathology, University Hospital Carl Gustav Carus, Technische Universität Dresden, Dresden, Germany; 5 Department of Neuropathology, Charité-Universitätsmedizin Berlin, Corporate Member of Freie Universität Berlin and Humboldt-Universität zu Berlin, Charitéplatz 1, Berlin, Germany; 6 Institute of Diagnostic and Interventional Neuroradiology, Faculty of Medicine and University Hospital Carl Gustav Carus, Technische Universität Dresden, Dresden, Germany; 7 Medical Department I, University Hospital Carl Gustav Carus, Technische Universität Dresden, Dresden, Germany; 8 National Center for Tumor Diseases (NCT) Dresden, a partnership between German Cancer Research Center (DKFZ), Faculty ofMedicine and University Hospital Carl Gustav Carus at TU Dresden, and Helmholtz-Zentrum Dresden - Rossendorf (HZDR), Dresden, Germany; 9 Department of Neuropathology, DGNN Brain Tumor Reference Center, University of Bonn, Bonn, Germany; 10 Skin Cancer Center at the University Cancer Center, National Center for Tumor Diseases (NCT/UCC), Dresden, Germany; 11 Department of Pathology, University Medicine Greifswald, Greifswald, Germany

**Keywords:** Epithelioid glioblastoma, BRAF mutation, MAPK inhibitors, Leptomeningeal disease

## Abstract

Epithelioid glioblastoma (eGB), a very aggressive and rare brain tumour, is associated with a dismal median overall survival. Effective therapies for patients with eGB, particularly with leptomeningeal dissemination, are still lacking. Here, we describe a case of a 25-year-old male diagnosed with an intramedullary cervical tumour with subsequent leptomeningeal disease. Histopathology identified a highly necrotising, epithelioid-type tumour with high cell density, most compatible with the diagnosis of an eGB. DNA analysis revealed an unprecedented B-Raf protooncogene, serine/threonine kinase (BRAF) gene variant in exon 15 (ENST00000288602.6, c.1799_1810delinsATG, p.(V600_W604delinsDG)), triggering activation of the mitogen-activated protein kinase (MAPK) pathway. Consequently, we initiated MAPK inhibitor (MAPKi) therapy, utilizing a combination of BRAF and mitogen-activated protein kinase kinase (MEK) inhibitors. Liquid chromatography–tandem mass spectrometry analysis confirmed the drugs’ presence in the patient’s cerebrospinal fluid, indicating their capacity to cross the blood-brain barrier. Remarkably, the patient responded very well to therapy and transitioned from a near-comatose state to significantly improved health, sustained for over three months. This study highlights that MAPKi, particularly targeted towards novel BRAFV600 mutations, might offer promising advancements in eGB treatment strategies.

**Supplementary Information:**

The online version contains supplementary material available at 10.1186/s40478-024-01834-8.

## Introduction

Epithelioid glioblastoma (eGB) is a rare but highly aggressive subtype of glioblastoma (GB) with a poor median overall survival (OS) of only a few months [1–3]. Recognised as a distinct subtype of GB, isocitrate dehydrogenase (IDH)-wildtype in WHO Classification of Tumors of the Central Nervous System (CNS) [4, 5], eGB is characterised by epithelioid cells with abundant cytoplasm, eccentrically located nuclei and prominent nucleoli [4]. Given the absence of specific molecular and immunohistochemical markers, diagnosis can often be challenging. From a molecular perspective, eGB harbour homozygous deletions in cyclin dependent kinase inhibitor 2 A/2B (*CDKN2A/2B*) and exhibits frequent mutations in the promotor region of the telomerase reverse transcriptase (*TERT*). Moreover, activating mutations in the B-Raf protooncogene, serine/threonine kinase (*BRAF*) are also common in this GB subtype [6–9].

Activation of BRAF leads to the activation of the mitogen-activated protein kinase (MAPK) pathway and, as such, to phosphorylation of mitogen-activated protein kinase kinase (MEK), which in turn causes activation of downstream mitogen-activated protein kinases such as extracellular-signal regulated kinase (ERK). Because the pathway co-regulates cell proliferation and survival, it is often constitutively activated in tumours through activating mutations in the *RAF* or *RAS* genes. Activating *BRAF*^*V600E*^ mutations can be targeted with BRAF inhibitors, which specifically bind to the *BRAF*^*V600E*^-mutant form of BRAF, combined with MEK inhibitors. It has been shown that tumours with mutations at the *BRAF*^*V600*^ locus other than V600E/K can also be treated with MAPK inhibitors (MAPKi), but the efficacy appears to be lower than for tumours with the V600E mutation [10]. The combined BRAF/MEK-inhibitor therapy was first implemented in *BRAF*^*V600E*^-mutant, malignant melanoma (MM), but several groups also reported on patients with *BRAF*^*V600E*^–mutant gliomas or eGB who have responded to MAPKi [11–16].

While leptomeningeal disease (LMD) is relatively rare, its incidence is steadily increasing, a trend explained by advances in oncological diagnosis and treatment [17]. Despite this, LMD represents an unmet medical need, with limited therapeutic options leading to poor prognosis. A recent large study involving 178 LMD patients reported that with the current standard therapies, OS is typically limited to few weeks or months [18]. However, there have been encouraging case reports demonstrating OS rates exceeding 11 months [19–21]. Regarding eGB, this subtype appears to contribute more frequently to LMD development compared to other GB forms [22].

Here, we present a case of eGB with LMD harbouring a novel *BRAF* p.(V600_W604delinsDG) mutation, displaying an active MAPK pathway that could be successfully targeted with MAPKi therapy.

## Methods

### Patient samples

For histological, immunohistochemical (IHC) and DNA analyses, we utilised formalin-fixed paraffin-embedded (FFPE) tumour tissue, obtained from the patient. The histological diagnosis was conducted by two independent consultant neuropathologists (MM and JR). Additionally, to facilitate mutational and mass spectrometry analyses, we collected CSF via external ventricular drain (EVD) and serum from the patient at corresponding points of time. The use of patient-derived material for these molecular analyses was approved by the ethics committee of the University of Dresden (EK118042018, EK539112021). Studies were conducted in accordance with the Declaration of Helsinki.

### Mutational analysis using a custom-designed Qiagen panel

The tumour area was marked on a haematoxylin-eosin (H&E) stained slide by a board-certified pathologist, and corresponding tumour areas were macrodissected from the FFPE block. Genomic DNA was extracted from the collected tumour material using the QIAamp DNA Mini Kit (Qiagen, Hilden, Germany). DNA concentration was quantified using the Qubit™ dsDNA BR Assay (Life Technologies Europe, Bleiswijk, Netherlands).

Analogous to the methodology described in Juratli et al. [23], we used a custom-designed amplikon panel from Qiagen to amplify mutation hotspots of genes (in case of point mutation) or whole genes (in case of loss of function) of *AKT1*, *ATRX*, *BRAF*, *CDKN2A*, *CIC*, *DAXX*, *EGFR*, *GNA11*, *GNAQ*, *H3F3A*, *H3F3B*, *IDH1*, *IDH2*, *KDM6A*, *KLF4*, *NF1*, *NF2*, *PIK3CA*, *PIK3R1*, *POLR2A*, *PTEN*, *SMARCB1*, *SMO*, *STAG2*, *SUFU*, *TP53*, *TRAF7*, and *TERT* promoter. Briefly, amplification was performed according to the protocol “QIAseq targeted DNA panel, May 2017” (Qiagen), followed by paired end next generation sequencing (2 × 200 bp) using the Illumina MiSeq platform (Illumina, San Diego, USA). Sequences were then analysed with the Qiagen CLC Genomics Workbench using HG19 as a reference genome and a customized analysis algorithm (coverage ≥ 200, allele frequency ≥ 5%).

### Immunohistochemistry

Tumour tissue was fixed in 4% formalin, embedded in paraffin, cut in 1–3 μm serial sections and dried for 30 min at 70 °C. Deparaffinization and immunostaining was performed using the BenchMark XT automated stainer (Ventana Medical Systems Inc., Oro Valley, USA) or Leica Bond III (Leica Biosystems, Germany) automated stainer. For BenchMark XT, the sections were treated with cell conditioning 1 (CC1) buffer (#950 − 124, Ventana) for 1 h. This was followed by an incubation with an anti-Epithelial Membrane Antigen (EMA) antibody (#M0613, Agilent Technologies, Santa Clara, USA, 1:200 dilution, 28 min incubation), an anti-Ki67 antibody (#M7240, clone MIB-1, Agilent Technologies, Santa Clara, USA, 1:50 dilution, 28 min incubation), an anti-Glial Fibrillary Acidic Protein (GFAP) antibody (#M0761, clone 6F2, Agilent Technologies, Santa Clara, USA, 1:200 dilution, 28 min incubation), an anti-ERK antibody (#9102, Cell Signaling, Danvers, USA, 1:50 dilution, 60 min incubation) or an anti-pERK antibody (#4376, Cell Signaling, 1:200 dilution, 2 h incubation). Bound antibodies were then visualized with the UltraView Universal Alkaline Phosphatase Red IHC Detection Kit (#760 − 501, Ventana) or the UltraView Universal DAB Detection Kit (#760 − 500, Ventana), followed by dehydration and mounting of the slides. Hematoxylin and eosin (HE) staining was performed with the Sakura Tissue Tek Prisma automated stainer (Tokyo, Japan), using a 1:2 mix from MERCK (#109249, Darmstadt, Germany) and SAV (FSTL-HL-2500-M-1, Flintsbach am Inn, Germany). For Leica Bond III, SMARCB1/INI1 staining was performed with Epitope Retrieval Solution 2 (#AR9640, Leica Biosystems, Germany) for 30 min, followed by incubation with an anti-INI1 antibody (#MSK078-05, clone MRQ-27, Zytomed Systems, Berlin, Germany, 1:100 dilution, 30 min incubation). Staining to detect H3K27me3 alterations was initiated with Epitope Retrieval Solution 1 (#AR9961, Leica Biosystems, Germany) for 30 min, followed by incubation with an anti-Tri-Methyl-Histone H3 (Lys27) antibody (#9733, clone C36B11, Cell Signaling, Danvers, USA, 1:400 dilution, 30 min incubation). For S100 staining, sections were treated with protease for 10 min. This was followed by an incubation with an anti-S100 antibody (#Z0311, Agilent Technologies, Santa Clara, USA, 1:200 dilution, 32 min incubation).

Slides stained with HE, ERK and pERK were scanned at 200x magnification using the Panoramic Scan II (3DHistech, Budapest, Hungary) and visualized with the CaseViewer v2.3 (3DHistech). Images were taken at 100x magnification. Stained slides with EMA, Ki67, GFAP, S100, SMARCB1/INI1, Tri-Methyl-Histone H3 (Lys27) were photographed with a DS-Fi3 colour camera (Nikon Corporation, Tokyo, Japan) and edited with NIS-Elements D 5.42.04. Images were taken at 200x and 400x magnification. All images were processed for white balance using Affinity Photo (Affinity, Nottingham, UK).

### Fluorescence in situ hybridisation

CDKN2A fluorescence in situ hybridisation (FISH) analysis was performed using the ZytoLight SPEC CDKN2A/CEN 9 Dual Color Probe (#Z-2063, ZytoVision, Bremerhaven, Germany). A 4 μm thick FFPE tissue section was mounted on StarFrost adhesive slides (Waldemar Knittel Glasbearbeitungs GmbH, Braunschweig, Germany) and air-dried overnight. Slides were baked at 70 °C for at least 10 min prior to hybridisation.

Further FFPE tissue section pretreatment was performed using the ZytoLight FISH-Tissue Implementation Kit (#Z-2028-20, ZytoVision, Bremerhaven, Germany) according to standard operating procedures. This included incubation in PT1 solution at 98 °C for 15 min, followed by treatment with pepsin solution for 2 min at 37 °C. Afterwards, tissue denaturation was performed at 75 °C for 10 min together with the probe, followed by hybridisation over night at 37 °C.

After hybridisation, the slides were mounted with DAPI/DuraTect Solution (ultra) (#MT-0008-0.8, ZytoVision, Bremerhaven, Germany). The entire tumour area was marked by a pathologist and from this area around 50 tumour cells from multiple views were examined for their fluorescent signals. Manual counting of orange (classical satellite III region D9Z3 of chromosome 9 / CEN9) and green (CDKN2A) signals was performed using a Zeiss fluorescence microscope and the OCULAR software was used for signal documentation (two-colour channel/red-green filter). The percentage of cells with homozygous (no green signal per cell) and heterozygous deletion (only one green signal per cell) was reported.

### Determination of BRAF/MEK inhibitors concentration in plasma and CSF of patients by liquid chromatography–tandem mass spectrometry

Sample preparation of plasma and CSF samples was different. Twenty microliters of plasma were diluted with 180 µL of water and mixed with 200 µL formic acid (1%). An automated solid phase extraction with an ASPEC XL (Gilson, Middleton, USA) and Oasis HLB (Waters, Milford, USA) cartridges were used. The cartridges were conditioned, loaded, washed and finally eluted with 1 mL methanol. The SPE extracts were evaporated to dryness and reconstituted with 200 µL. For quantification, 10-point external calibration curves in blank plasma at the range from 1.95 to 1000 µg/L for all four substances were freshly prepared and measured in parallel.

Native CSF samples were used with a standard addition method for quantification. A volume of 20 µl was always injected into the Ultimate 3000 HPLC system from Thermo Scientific system coupled to an API4000 tandem mass spectrometer (ABSciex, Darmstadt, Germany). The BRAF and MEK inhibitors encorafenib, binimetinib, dabrafenib and trametinib were separated by reversed phase chromatography with a Synergi 4 μm Hydro-RP 80 A, 150 × 3 mm column (Phenomenex, Torrance, USA).

The temperature of column was constantly at 40 °C. Determination of the analytes was performed using the multiple reaction monitoring mode with nitrogen as collision gas. For positive ionization, a capillary voltage of 4000 V (positive ion mode) was used. Quantification was performed by the peak area method, and a weighted (1/x) regression of first order yielded the concentrations.

### Extraction and quantification of cell-free DNA

Cell-free DNA (cfDNA) was extracted from the CSF using the QIAamp Circulating NA Kit (Qiagen, Hilden, Germany) according to manufacturer’s instructions. cfDNA was eluted into 60 µL TE buffer, quantified by a β-globin-specific qPCR in comparison to a serial dilution of a reference DNA with known quantity on a 7500 Real-Time PCR System (Applied Biosystems, Foster City, CA, USA) and stored at -20 °C until downstream processing for NGS.

### NGS analysis for the detection of cfDNA

cfDNA samples were analysed according to an optimized protocol for error-reduced NGS-based detection of low-level single nucleotide variants (SNVs), as described previously [24]. Briefly, fusion PCR primers for the preparation of amplicon libraries were designed (Primer Premier 6; Premier Biosoft, Palo Alto, CA, USA) according to the manufacturer’s recommendations (Fusion Method; Life Technologies). PCR amplification of the *BRAF* gene was performed using the Q5^®^ High-Fidelity proofreading polymerases (New England Biolabs, Beverly, MA, USA) and primer sequences 5′-CCTTTACTTACTACACCTCAGA-3′ (Forward) and 5′-GATCCAGACAACTGTTCAAACT-3′ (Reverse) with amplification thermocycle consisting of: 98 °C for 30 s, followed by 40 cycles at 98 °C for 5 s, 61 °C for 10 s, and 72 °C for 20 s, final extension at 72 °C for 2 min. In order to increase sensitivity and specificity for low-level cfDNA, PCR of individual samples was performed in triplicate separate reactions (using different molecular barcodes for binning). After Fusion PCR on a GeneAmp PCR System 9700 (Applied Biosystems), purified and barcoded PCR products were diluted to 30 pM and loaded on an Ion Chef instrument (Life Technologies) for automated template preparation. Deep sequencing (aiming for > 100.000fold coverage) was conducted on an Ion S5 XL NGS system (Life Technologies). Raw data alignment of demultiplexed FastQ files, variant calling and filtering was done using the Sequence Pilot software package (JSI medical systems GmbH, Ettenheim, Germany) with default settings. Human genome build HG19 (http://genome.ucsc.edu/) was used as reference genome for mapping algorithms. Based on mutation-specific false-positive error rates, detection of cfDNA was conducted with a sensitivity of 0.01 VAF. VAFs below the predefined threshold were considered cfDNA negative.

### 850k methylation bead arrays

Genome-wide methylation profiles were generated via the Infinium MethylationEPIC (850 k) BeadChip array (Illumina, San Diego, USA). The Heidelberg brain classifier version 12.8 was used via the website https://www.molecularneuropathology.org to annotate the profiles to a methylation class; scores > 0.9 were considered as match.

## Case description and results

A 25-year-old male patient presented with progressive tingling paresthesia of the right foot and hand. Despite intravenous cortisone administration, his symptoms persisted and were coupled with neck pain radiating to the shoulders. An MRI of the entire spine and neurocranium revealed a contrast-enhancing spinal lesion at levels C3–7 with cystic formations, yet no neurocranium abnormalities. Subsequently, he was referred to our neurosurgery department for tumour resection. Surgery was performed via a laminotomy of C3–7, revealing a highly vascularized and partially necrotic tumour that was poorly demarcated from the spinal cord, resulting in only subtotal tumour resection.

**Fig. 1:**
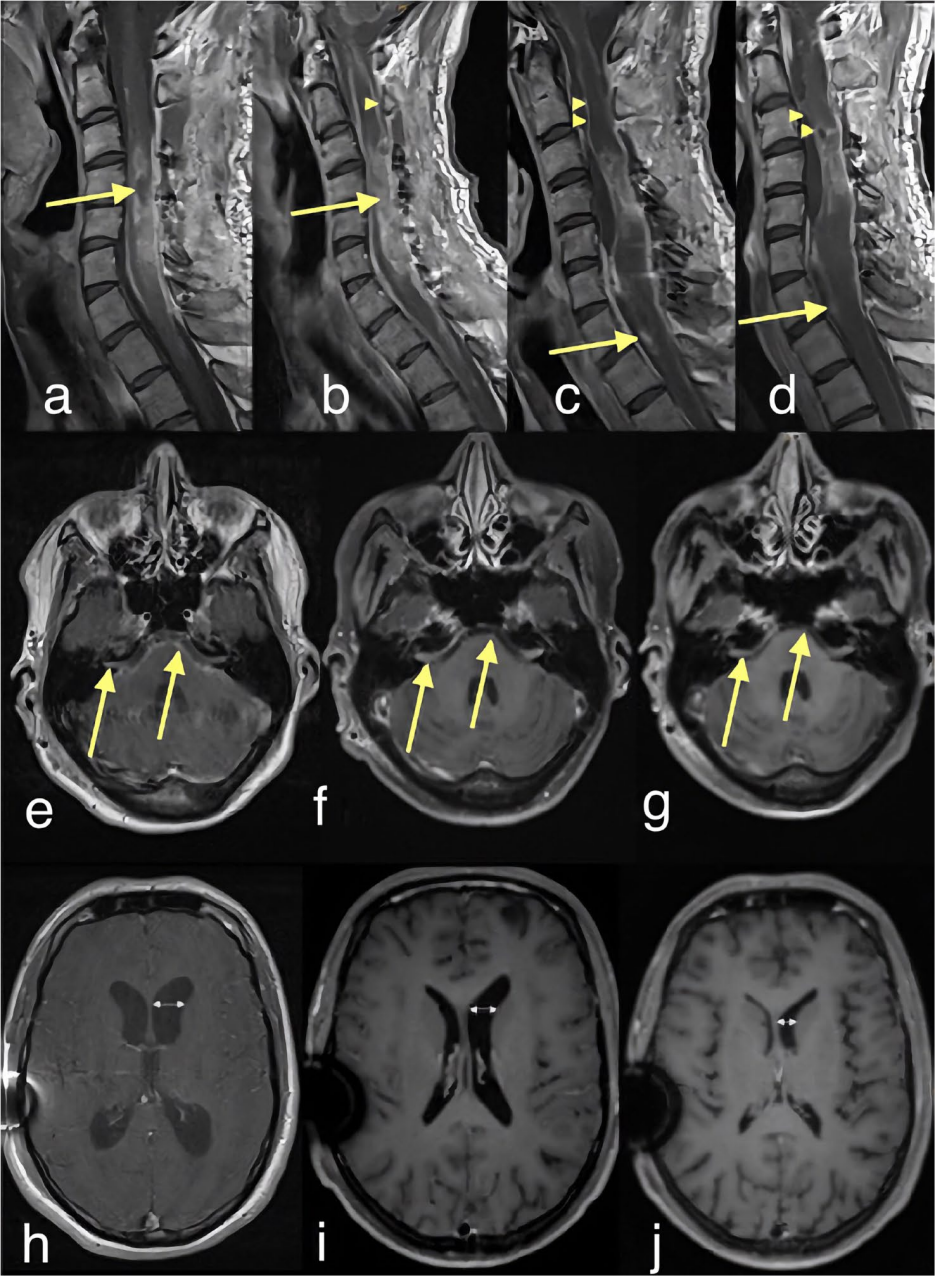
Contrast-enhanced T1w magnetic resonance imaging (MRI) of the cervical myelon (**a**-**d**) and neurocranium (**e**-**j**). Row **a**-**d** show the course of spinal involvement: **b** depicts tumour progression with leptomeningeal (arrowhead) and parenchymal involvement (arrow) within 18 days. Subsequently, the therapy was initiated. **c** and **d** show regression of contrast enhancement within 15 days (**c**) and after 25 days (**d**). **e**-**g** show regression of intracranial involvement with leptomeningeal contrast enhancement of the pons and cranial nerves VII and VIII. **h**-**j** demonstrate decrease in ventricular width after therapy initiation and shunt implantation

Approximately two weeks post-surgery, the patient complained of severe occipital headaches. An emergency CT scan showed a hydrocephalus occlusus, leading to the placement of an EVD, later replaced by a ventriculoperitoneal shunt (VPS). Six days later, the patient returned with deteriorating headaches, nausea and drowsiness. A subsequent MRI showed an extensive localised intramedullary tumour recurrence from C3 to C6/7, causing severe narrowing of the spinal canal and additional evidence of LMD, which was prominent around the brainstem and spinal cord (Fig. 1a-b, e and h). Furthermore, laboratory analyses of the CSF revealed highly elevated protein levels and a highly elevated cell count (Table 1), prompting a decision for repeated tumour mass debulking.

With the patient’s condition progressively deteriorating, marked by increased somnolence and neurological deficits, a new EVD was placed, as the existing VPS proved insufficient due to vastly increased cell count. The prospect of transitioning to best supportive care was discussed at this stage [Eastern Cooperative Oncology Group (ECOG) Score 4, Karnofsky Performance Status (KPS) 10–20%].

**Table 1:** Cerebrospinal fluid laboratory results before and during treatment with BRAF/MEK inhibitors

	Protein level	Cell count
**Normal**	50–150 mg/L	< 2 MPt/L
**Before therapy**	1283 mg/L	112 MPt/L
**Under therapy**	493 mg/L	71 MPt/L

**Fig. 2:**
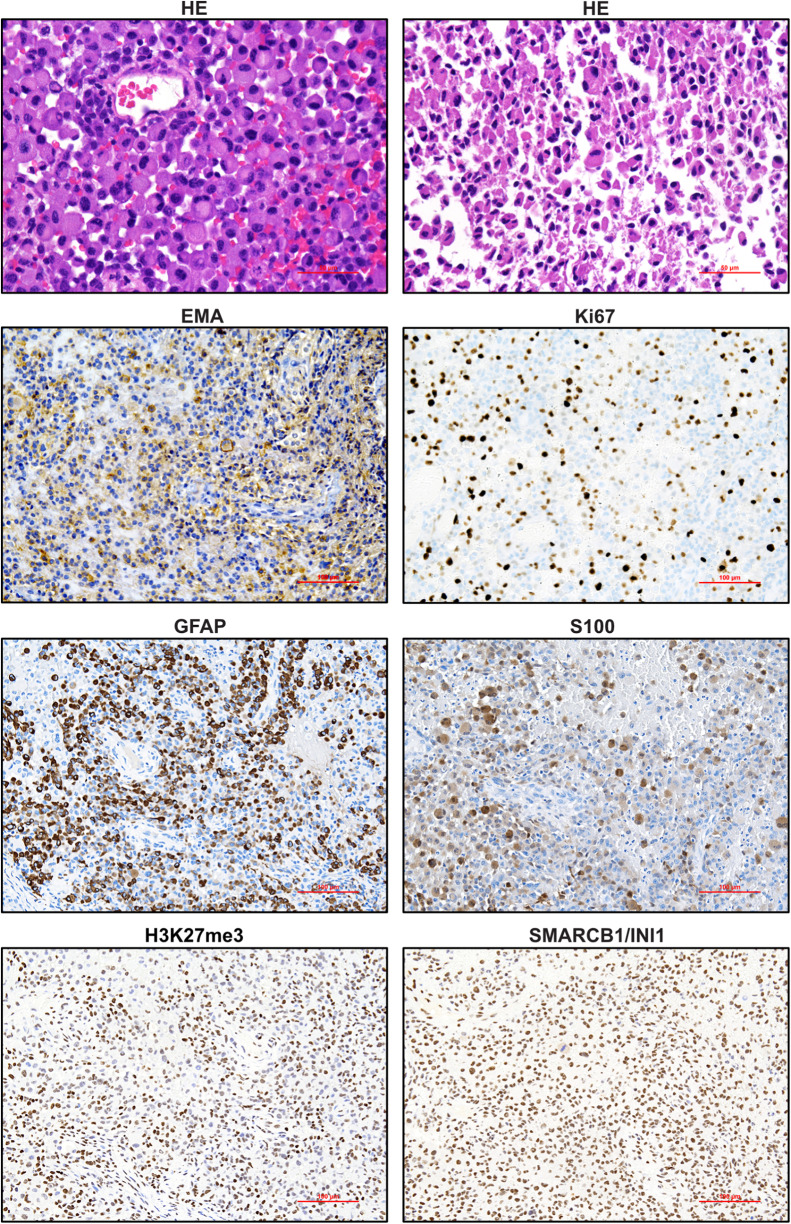
Immunohistochemistry analysis of epithelioid glioblastoma. FFPE tissue was stained with HE or with antibodies against EMA, Ki67, GFAP, S100, H3K28me3 and SMARCB1/INI1. A representative area at a magnification x 200 or x 400 (for HE) is shown, scale bar indicates 100–50 μm, respectively

In order to better characterise the tumour and derive an effective treatment strategy, we conducted extensive molecular and IHC analyses on the patient’s tumour tissue (Fig. 2). Histopathology revealed a malignant, highly necrotizing, epithelioid tumour densely packed with cells, most of which exhibited a large cytoplasm with clear cell borders, along with eccentric nuclei. Glial fibres, characteristic glial matrix or cell processes were not clearly recognisable, which initially prompted considerations for differential diagnosis such as malignant melanoma (MM) or rhabdoid meningioma. Nevertheless, the patient’s age, focal but significant GFAP expression, and the intramedullary tumour growth raised the possibility of H3 K28 (K27)-altered diffuse midline glioma. However, we found no evidence of loss of nuclear staining in the tumour nuclei for H3 p.K28me3 (K27me3; Fig. 2) as well as for the glial marker Olig2 (data not shown). The DNA methylation profile generated using the Infinium MethylationEPIC BeadChip Kit, as previously described [25], showed a significant calibrated score of 0.93 for methylation class GB, IDH-wildtype, mesenchymal subtype (subclass B) in the Heidelberg brain classifier version 12.8 (Supplementary Fig. 1, Additional file 1). This subclass is provisional but appears to show a certain enrichment for eGB.

An additional t-distributed stochastic neighbour embedding (t-SNE) analysis of the methylation profile confirmed the similarity to IDH-wildtype GB (Supplementary Fig. 2, Additional file 1). FISH analysis was used to validate the homozygous loss of the *CDKN2A* locus (Supplementary Fig. 3, Additional file 1). Taking all findings into account and in line with the current scientific literature [26–28], we diagnosed an eGB.

Using a custom-designed next generation sequencing panel, we investigated alterations in brain tumour-relevant genes responsible for various cellular functions such as signalling and growth regulation (*AKT1*, *BRAF*, *EGFR*, *GNA11*,* GNAQ*, *NF1*, *NF2*,* PIK3CA*, *PIK3R1*,* PTEN)*, differentiation (*SMO*,* SUFU*), apoptosis (*TRAF7*), cell cycle regulation (*CDKN2A*, *TP53*), energy regulation (*IDH1*, *IDH2*) as well as DNA modification and transcription (*ATRX*, *CIC*, *DAXX*, *H3F3A*, *H3F3B*,* KDM6A*, *KLF4*,* SMARCB1*, *STAG2*, *TERT* promotor) [23]. In line with the findings above, the panel sequencing revealed a loss of *CDKN2A* and wildtype sequences for *IDH-1*, *IDH-2* and *SMARCB1.* All other tested genes, apart from the *BRAF* gene also displayed wildtype sequences. Within the *BRAF* gene, we identified a novel mutation in exon 15 (c.1799_1810delinsATG, p.(V600_W604delinsDG)). The same *BRAF* mutation was detectable in cfDNA extracted from CSF, with a variant allele fraction (VAF) of 2.036% at a coverage of 2,366,563 reads.

**Fig. 3:**
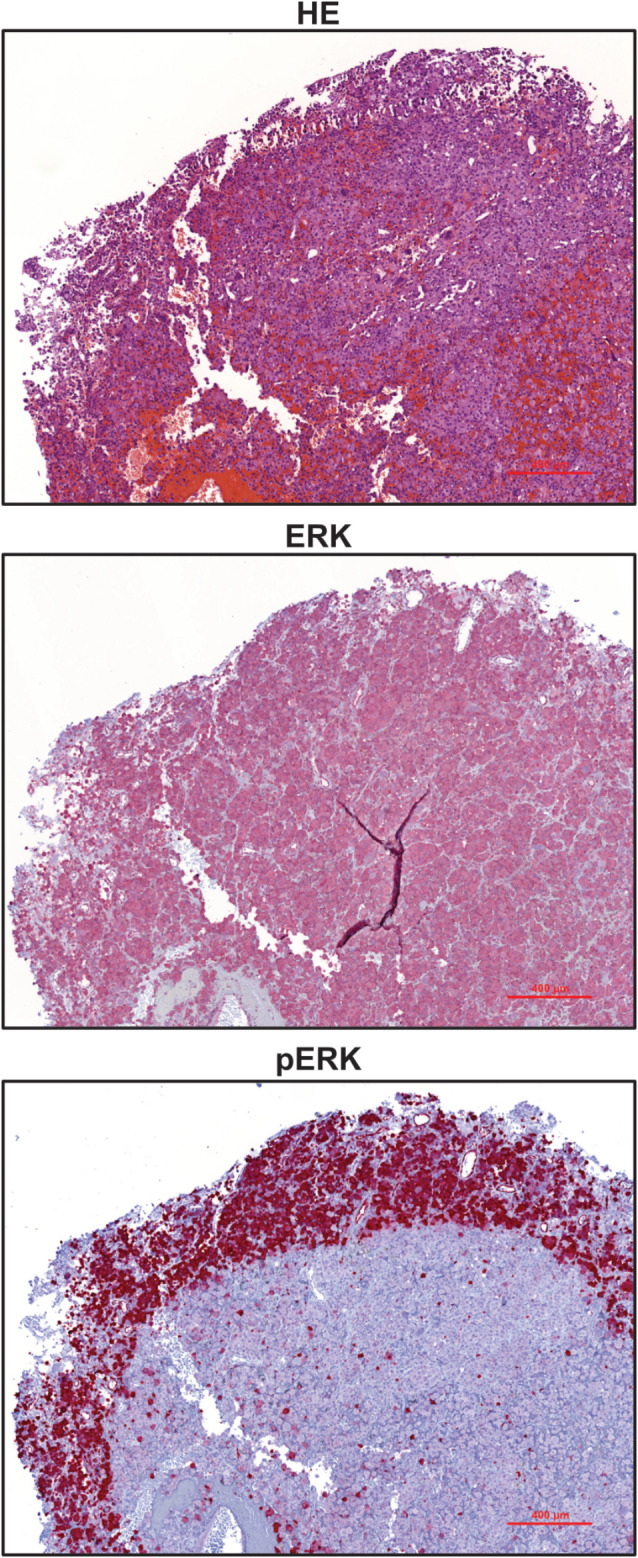
Immunohistochemistry analyses reveal activation of the MAPK pathway. FFPE tissue of the case was stained with HE or using antibodies against ERK and phospho-ERK (pERK). A representative area at a magnification x 100 is shown. Scale bar indicates 100 μm

Because it is unclear whether this novel *BRAF* mutation causes activation of the MAPK pathway, we conducted IHC on our patient’s tumour tissue. Intriguingly, our results showed uniform expression of ERK throughout the tumour and strong expression of its activated form, pERK, at the tumour edges, verifying that the novel *BRAF* mutation causes constitutively activated MAPK signalling (Fig. 3).

Based on the promising results of the mutational and IHC analyses, a targeted therapy with the BRAF inhibitor, encorafenib and the MEK inhibitor, binimetinib was initiated. The dosage mirrored that of MM with 450 mg of encorafenib (i.e. 6 × 75 mg) administrated daily, and 45 mg of binimetinib (i.e. 3 × 15 mg), administrated twice daily. Despite a slight improvement in his general condition post-initiation of the treatment, the quantity and the size of the hard capsules and tablets were poorly tolerated by the patient. Consequently, after three days, the regimen was modified. An alternative targeted therapy with the BRAF inhibitor, dabrafenib at a dosage of 150 mg (i.e. 2 × 75 mg) administrated twice daily, and the MEK inhibitor, trametinib at 2 mg daily, was introduced and proved to be better tolerated by the patient.

Moreover, comprehensive analyses of the patients’ CSF and plasma using liquid chromatography-tandem mass spectrometry were performed to determine the concentrations of various MAPKi two, four, and five days after regimen modification, i.e. five, seven, and eight days following initiation of the targeted therapy. There was a clear accumulation of all MAPKi in the patient’s plasma (Table 2). The concentrations determined reside within the therapeutic range of the maximum and minimum plasma concentrations published (Table 2). Interestingly, even though the concentrations were lower, all MAPKi could also be detected in the patient’s CSF (Table 2), indicating that MAPKi can cross the blood-brain-barrier and, as such, may influence tumour growth.

**Table 2:** Dosage and concentration of dabrafenib, trametinib, encorafenib and binimetinib measured in the cerebrospinal fluid (CSF) and serum after different time points

Medication	Dabrafenib			Trametinib			Encorafenib			Binimetinib		
**Dosage (mg/d)**	300 (2 × 150)			2 (1x)			450 (1x)			90 (2 × 45)		
**Day**	5	7	8	5	7	8	5	7	8	5	7	8
**Serum (ng/ml)**	322.9	188.0	94.6	8.8	7.7	9.2	13.5	5.7	6.1	96.5	19.1	13.8
**CSF (ng/ml)**	5.4	6.1	1.5	0.0	0.3	0.4	2.4	1.1	1.1	10.3	5.3	5.0
**C**_**max**_ **(ng/ml)**	1478			22.2			3800			654		
**C**_**min**_ **(ng/ml)**	26			12.1			13.9			51.4		
**CSF/Serum (%)**	1.7	3.2	1.6	0.0	3.9	4.3	17.8	19.3	18.0	10.7	27.7	36.2

Encorafenib and binimetinib were administered at day 1–3 and then discontinued. Dabrafenib and trametinib were started at day 4. Serum and CSF samples were taken at 5, 7 and 8 days after treatment initiation. C_max_ (maximum plasma concentration) and C_min_ (minimum plasma concentration) of BRAFi and MEKi measured in the serum of patients was obtained from the product information sheet at the European Medicines Agency (https://www.ema.europa.eu/en, accessed on 6th July 2023) or from Delord et al., 2017 [29] and Sullivan et al., 2020 [30]. With exception of encorafenib (86%), all medications demonstrate a high binding to plasma proteins (> 97%) according to SmPC

Within the first week of targeted therapy, the patient experienced a significant improvement in alertness. In addition, the patient improved mobility in a very short time (ECOG 2, KPS 50–60%). With the assistance of physical therapists, he regained the ability to walk with a rollator and, with the aid of a nurse, managed to independently shower and dress. Likewise, the parameters (cell count and protein) collected via the EVD demonstrated improvement (Table 1). An MRI of both the brain and spinal axis, conducted 10 days post- therapy initiation, revealed a decrease in ventricular width and leptomeningeal enhancement, as along with a partial regression of the contrast agent uptake and the space-occupying effect of the intramedullary tumour (Fig. 1c-d, f-g and l-j). While he was still in the process of regaining strength during his time in our clinic, he made enough progress to be transferred to a rehabilitation facility for further recovery.

Three months after beginning with the targeted therapy, the patient was readmitted with recurring episodes of hallucinations and confusion. The MRI scan showed massive local tumour recurrence and progressive LMD. As previously anticipated, the tumour had become resistant to the targeted therapy, leading to its discontinuation. At this point, no other therapeutic options were available for the patient, thus best supportive care was agreed upon. The patient passed away within two weeks.

In summary, the patient initially recovered from a semi-conscious state and survived for more than three months post-therapy initiation. This suggests that MAPKi present substantial benefits for patients with advanced *BRAF*-mutant eGB.

## Discussion

In this case report, we discuss a patient with eGB who experienced an escalating deterioration and emergent somnolence due to tumour progression and the development of LMD. A mutational analysis on FFPE tumour tissue and cfDNA extracted from CSF unveiled a unique variant in exon 15 of *BRAF*, a variant never before identified in this entity. Through IHC analysis, we determined an active MAPK pathway, guiding us towards a treatment strategy. Subsequent initiation of treatment with MAPKi, detectable in the patient’s serum and CSF, provided significant clinical benefit.

High-grade gliomas, including classic GB, seldom have *BRAF* mutations [31] and yield poor therapeutic outcome due to the low mutation rate [32]. In contrast, 50–93% of eGB harbour *BRAF*^*V600E*^ mutations [6, 9] making them suitable for MAPKi targeted therapy. Several groups reported successful responses to MAPKi in patients with *BRAF*^*V600E*^–mutant gliomas or eGB [11–16]. A Japanese group reported a similar case where rapid clinical and radiological response were observed following dabrafenib- and trametinib treatment in a patient with a *BRAF*^*V600E*^-mutant eGB [33]. Furthermore, the authors successfully established a cell line containing the *BRAF*^*V600E*^ mutation, *TERT* promoter mutation and *CDKN2A/2B* loss from recurrent tumour derived from an autopsy. Intracranial implantation of these cells into mice resulted in tumours quite similar to the original and also responded well to MAPKi therapy.

In the current study, our eGB patient presented a novel *BRAF*^*V600*^ mutation p.(V600_W604delinsDG), rather than a conventional *BRAF*^*V600E*^ mutation. The COSMIC database lists three entries with the same amino acid deletions (V600-W604) but different nucleotide alterations. These mutations were identified in two ganglioglioma and one papillary thyroid cancer [34–36]. This includes one patient with desmoplastic infantile ganglioglioma, who responded well to MAPKi [35]. Our IHC data displaying an active MAPK pathway, along with the clinical response of our eGB patient to MAPKi, clearly supports an oncogenic role for the newly identified *BRAF*^*V600*^ mutation.

While MAPKi are known to cross the blood-brain barrier (BBB), the extent to which these drugs reach therapeutic concentrations remains unclear [37]. Data from this study reaffirms the ability of MAPKi to penetrate the BBB. The patient’s notable clinical improvement suggests that MAPKi reach therapeutic concentrations in the CNS, at least for a limited duration. However, CNS levels are clearly lower than serum levels in our eGB patient (Table 2). Evidence indicates that BRAF inhibitors may have limited access to the brain and leptomeningeal lesions due to active drug efflux transporters [38]. In addition, the restricted BBB penetration of MAPKi to the meningeal space might be due to their relatively high molecular weight and poor lipid solubility [39]. In a case series of six individuals, significant inter-individual differences in CSF concentrations of the BRAF inhibitor vemurafenib were observed [37]. The variability in plasma and CSF levels in these patients might be due to differences in the integrity of the BBB, particularly after prior local treatments such as surgery or radiotherapy. This highlights the urgent need for further investigation and the development of MAPKi with reliable CNS penetration. Prospective studies are underway, for instance, a phase 1 trial of a novel CNS-penetrant BRAF inhibitor, PF-07284890, in combination with binimetinib in patients with *BRAF*^*V600*^-mutant solid tumours with or without CNS-involvement or LMD (NCT04543188). Moreover, preclinical data on a novel BRAF inhibitor (compound Ia) showed promising intracranial results due to limited P-glycoprotein-mediated efflux and a lower molecular weight, which may facilitate brain penetration [40].

Alongside the challenge of reaching the intended site, the overall response duration to MAPKi is typically limited by multiple mechanisms of acquired resistance [41]. In line with this, we assume that our patient also initially responded to treatment, but over time developed acquired resistance, leading to renewed tumour growth with no possibility of further therapy modality due to rapid advancement. Additionally, treating LMD presents considerable difficulty in general. Although the exact reasons for this remain incompletely understood, studies suggest a reprogramming of the LMD tumour microenvironment, characterised by a dysfunctional T cell landscape, which diminishes the efficacy of systemic therapy [42].

In terms of different therapy options, radiation was considered multiple times throughout the patient‘s treatment. Shortly bevor initiating radiation, we found the patient’s condition deteriorated for the tumour had already progressed. As the target volume would have been quite large, we opted for targeted therapy instead of radiation. Even after discontinuation of MAPKi, initiating radiation and/or chemotherapy would not have provided a realistic chance of recovery. By that point, the patient’s overall condition had already deteriorated, with recurrent episodes of hallucinations suggesting he would likely not have tolerated further treatment well.

Regarding the location of the tumour, it must be said that the spine as the primary site is highly unusual. In this context, there is only one published case report on PubMed, which is an Indian publication reporting on an eGB in the conus medullaris [43]. Thus, we present a case that not only features a previously undescribed mutation but also exhibits an extremely unusual localisation, making it noteworthy in several respects.

## Conclusions

Rare tumours pose unique challenges in oncology due to their limited incidence and diverse characteristics. Traditional diagnostic and treatment approaches often fail to sufficiently address these tumours. Nevertheless, advances in molecular analysis have created new opportunities for understanding and treating these rare malignancies. Using NGS panel sequencing and liquid biopsy approaches, we identified a novel BRAF mutation within this case of eGB. We confirmed that the BRAF mutation activated the MAPK pathway using IHC, making it treatable with MAPKi. The presence of the inhibitors in the CSF was confirmed using liquid chromatography-tandem mass spectrometry. The therapy achieved a remarkable response. Prior to treatment, the patient’s performance status was ECOG 4/KPS 10–20%, i.e. the patient was somnolent, completely disabled, could not carry on any selfcare and was totally confined to bed. After starting treatment, the patient showed significant improvement: he was able to walk using a rollator, and with the assistance of a nurse, he could take a shower and dress himself (ECOG 2, KPS 50–60%).

Therefore, our approach emphasizes the importance of analysing rare tumours based on their molecular, cellular and functional characteristics, ultimately leading to the development of site-agnostic precision cancer medicine. However, deriving a general therapy scheme for underlying rare tumour types, particularly when there is only one known case so far, is notoriously difficult, and therefore a primary limitation of this work.

### Electronic supplementary material

Below is the link to the electronic supplementary material.

Supplementary Material 1

## Data Availability

Data sharing is not applicable to this article as no datasets were generated or analysed during the current study.

## Abbreviations

BBB: Blood-brain barrier
BRAF: B-Raf protooncogene, serine/threonine kinase
CDKN2A/2B: Cyclin dependent kinase inhibitor 2 A/2B
CIC: Capicua transcriptional repressor
cfDNA: Cell-free DNA
C_max_: maximum plasma concentration
C_min_: minimum plasma concentration
CNS: Central nervous system
CSF: Cerebrospinal fluid
CT: Computed tomography
d: Day
DAB: Ultraview universal dab detection kit
DKFZ: Deutsches Krebsforschungsinstitut = German Cancer Research Center
DNA: Desoxyribonucleic acid
eGB: Epithelioid glioblastoma
EGFR: Epidermal growth factor receptor
EMA: Epithelial membrane antigen
ERK: Extracellular-signal regulated kinase
EVD: External ventricular drain
FISH: Fluorescence in situ hybridisation
FFPE: Formalin-fixed paraffin-embedded
GB: Glioblastoma
GFAP: Glial fibrillary acidic protein
GNA11: G protein subunit alpha 11
GNAQ: G protein subunit alpha Q
HE: Haematoxylin-eosin
HZDR: Helmholtz Zentrum Dresden
IHC: Immunohistochemical
IDH: Isocitrate dehydrogenase
KLF4: Krüppel-like factor
LMD: Leptomeningeal disease
MAPK: Mitogen-activated protein kinase
MAPKi: Mitogen-activated protein kinase inhibitor
MEK: Mitogen-activated protein kinase kinase
mg: milligram
ml: milliliter
MM: Malignant melanoma
MRI: Magnetic resonance imaging
NCT: National Center for Tumor Diseases
NF1/2: Neurofibromatosis type 1/2
ng: nanogram
NGS: Next generation sequencing
OS: Overall survival
PCR: Polymerase chain reaction
pERK: phosphorylated extracellular-signal regulated kinase
PIK3CA: Phosphatidylinositol-4,5-bisphophate 3-kinase catalytic subunit alpha
PIK3R1: Phosphoinositide-3-kinase regulatory subunit 1
POLR2A: Polymerase (RNA) II (DNA directed) polypeptide A
PT1: Pretreatment solution citric
PTEN: Phosphatase and tensin homolog
RNA: Ribonucleic acid
SMARCB1/INI-1: SWI/SNF related matrix associated actin dependent regulator of chromatin, subfamily b, member 1
SmPC: Summary of product characteristics
SNV: Single nucleotide variant
SPE: Solid phase extraction
TERT: Telomerase reverse transcriptase
TERTp: Telomerase reverse transcriptase promoter
TNF: Tumor necrosis factor
TP53: Tumor protein p53
TRAF7: TNF receptor associated factor 7
t-SNE: t-distributed stochastic neighbour embedding
UCC: University Cancer Center
VAF: Variant allele frequency
VPS: Ventricularperitoneal shunt
WHO: World Health Organization
