# Supplementary material for: Efficacy of BRAF/MEK-inhibitor therapy for epithelioid glioblastoma with a novel BRAFV600 mutation
Source: Acta Neuropathol Commun. 2024 Aug 6;12:124. doi: 10.1186/s40478-024-01834-8 (PMC11302837; doi:10.1186/s40478-024-01834-8)
Supplement: Supplementary file 1 — Supplementary Material 1 [file 40478_2024_1834_MOESM1_ESM.pdf]

Supplementary figures

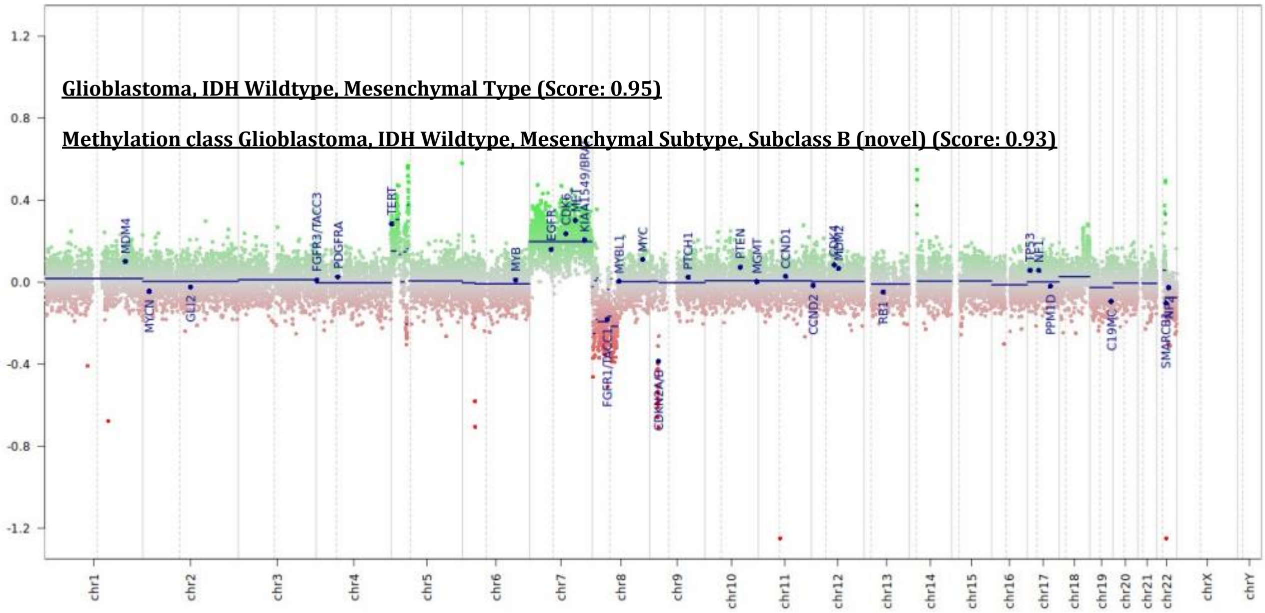

Supplementary figure 1. 850k methylation bead arrays. *Genome-wide DNA methylation analysis and copy number analysis demonstrated homozygous CDKN2A/B deletion and gain of chromosome 7. The O-6-methylguanine DNA methyltransferase (MGMT) promoter (MGMT-STP27) was methylated.*

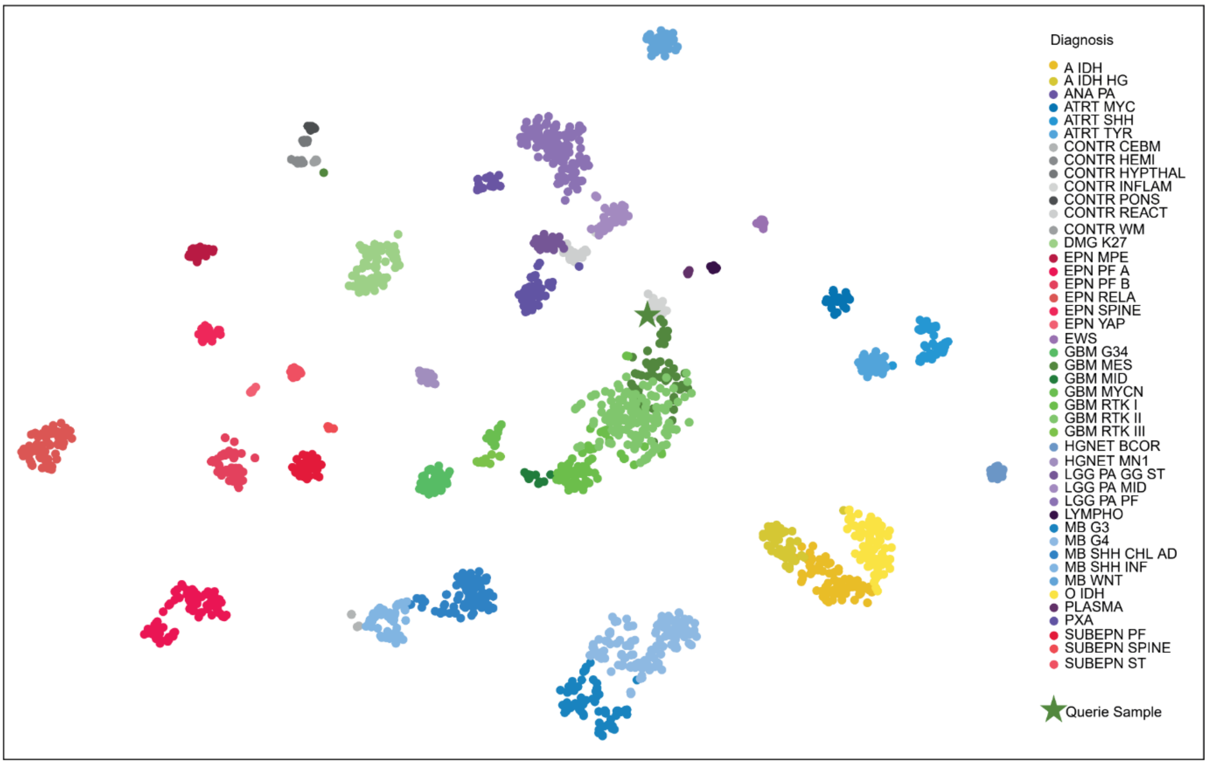

Supplementary figure 2. t-distributed stochastic neighbour embedding (t-SNE) of the present case together with selected DNA-methylation based reference classes taken from Capper et al. [1]. *The case clusters very close to the reference class of glioblastoma, IDH wildtype mesenchymal (GBM MES). A IDH - IDH glioma, subclass astrocytoma, A IDH HG - IDH glioma, subclass high grade astrocytoma, ANA PA - anaplastic pilocytic astrocytoma, ATRT MYC - atypical teratoid/rhabdoid tumor, subclass MYC, ATRT SHH - atypical teratoid/rhabdoid tumor, subclass SHH, ATRT TYR - atypical teratoid/rhabdoid tumor, subclass TYR, CONTR CEBM- control tissue, cerebellar hemisphere, CONTR HEMI - control tissue, hemispheric cortex, CONTR HYPTHAL - control tissue, hypothalamus, CONTR INFLAM - control tissue, inflammatory tumor microenvironment, CONTR PONS - control tissue, pons, CONTR REACT - control tissue, reactive tumor microenvironment, CONTR WM - control tissue, white matter, DMG K27 - diffuse midline glioma H3 K27M mutant, EPN MPE - ependymoma, myxopapillary, EPN PF A - ependymoma, posterior fossa, group A, EPN PF B - ependymoma, posterior fossa, group B, EPN RELA - ependymoma, RELA fusion, EPN SPINE - ependymoma, spinal, EPN YAP - ependymoma, YAP fusion, EWS - ewing sarcoma, GBM G34 - glioblastoma, IDH wildtype, H3.3 G34 mutant, GBM MES - glioblastoma, IDH wildtype, subclass mesenchymal, GBM MID - glioblastoma, IDH wildtype, subclass midline, GBM MYCN - glioblastoma, IDH wildtype, subclass MYCN, GBM RTK I - glioblastoma, IDH wildtype, subclass RTK I, GBM RTK II - glioblastoma, IDH wildtype, subclass RTK II, GBM RTK III - glioblastoma, IDH wildtype, subclass RTK III, HGNET BCOR - high grade neuroepithelial tumor with BCOR alteration, HGNET MN1 - high grade neuroepithelial tumor with MN1 alteration, LGG PA GG ST - low grade glioma, subclass hemispheric pilocytic astrocytoma and ganglioglioma, LGG PA MID - low grade glioma, subclass midline pilocytic astrocytoma, LGG PA PF - low grade glioma, subclass posterior fossa pilocytic astrocytoma, LYMPHO - lymphoma, MB G3 - medulloblastoma, subclass group 3, MB G4 - medulloblastoma, subclass group 4, MB SHH CHL AD - medulloblastoma, subclass SHH A (children and adult), MB SHH INF - medulloblastoma, subclass SHH B (infant), MB WNT - medulloblastoma, WNT, O IDH - IDH glioma, subclass 1p/19q codeleted oligodendroglioma, PLASMA - plasmacytoma, PXA - (anaplastic) pleomorphic xanthoastrocytoma, SUBEPN PF - subependymoma, posterior fossa, SUBEPN SPINE - subependymoma, spinal, SUBEPN ST - subependymoma, supratentorial*

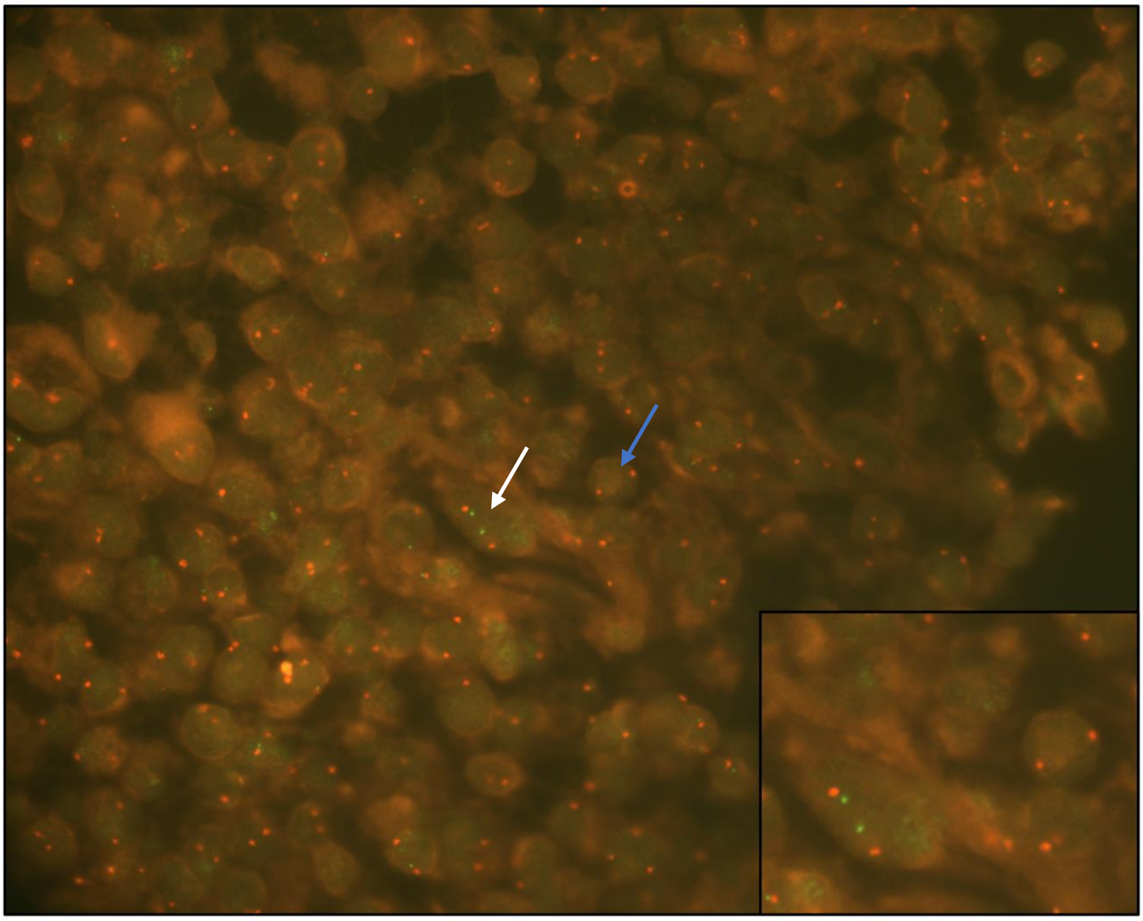

Supplementary figure 3. FISH analysis shows homozygous deletion of CDKN2A*. FFPE tissue was hybridised with a CDKN2A (green) / CEN 9 (orange) Probe over night at 37°C. Fifty tumour cells from different fields of view were examined for their fluorescent signals and the percentage of cells with homozygous CDKN2A deletion (no green signal per cell) was calculated. A representative image at a magnification x 200 (with insets in higher magnification) is displayed. White arrow indicates a cell with two copies of CDKN2A, the blue marks a cell with no copies of CDKN2A (homozygous deletion). Homozygous deletion of CDKN2A was found in 68% of the examined tumour cells.*

References

1. Capper D, Jones DTW, Sill M, Hovestadt V, Schrimpf D, Sturm D et al (2018) DNA methylation-based classification of central nervous system tumours. Nature 555:469–474
